# Development of a porcine training model for microvascular fasciocutaneous free flap reconstruction

**DOI:** 10.1186/s13005-024-00435-z

**Published:** 2024-06-03

**Authors:** Christopher-Philipp Nobis, Katharina Grottschreiber, Manuel Olmos, Tobias Moest, Manuel Weber, Marco Kesting, Rainer Lutz

**Affiliations:** Department of Oral and Cranio-Maxillofacial Surgery, University Hospital Erlangen, Friedrich-Alexander-Universität Erlangen-Nürnberg (FAU), Glueckstrasse 11, D-91054 Erlangen, Germany

**Keywords:** Surgical training model, Free flap surgery, Microvascular flap, Oral and maxillofacial surgery, Plastic and reconstructive surgery, Porcine animal model

## Abstract

**Background:**

In reconstructive surgery, improvements are needed in the effective teaching of free flap surgery. There is a need for easily accessible and widely available training without high financial costs or ethical concerns while still providing a realistic experience. Our aim was to develop an appropriate training model for microvascular flaps.

**Methods:**

We identified pig head halves as most appropriate regarding availability, cost, and realism. These accrue largely by the food industry, so no animals need to be sacrificed, making it more ethical from an animal welfare perspective. We evaluated the suitability as flap donor site and analyzed the vascular anatomy of 51 specimens.

**Results:**

Anatomical evaluation revealed a reliable and constant vascular anatomy, allowing the design of a flap model that can effectively illustrate the entire process of microvascular flap surgery. The process was divided into 6 key steps. The flap can be harvested after marking the vascular pedicle 5.3 cm from the lateral corner of the mouth. Skin island design and subsequent tissue dissection follow until a fasciocutaneous flap is raised, similar to a radial flap. Upon completion of flap harvesting, it can be freely transferred for defect reconstruction. Microvascular anastomosis can be performed on recipient vessels in the cervical region, and the difficulty can be individually adjusted.

**Conclusions:**

The developed training model is a reasonable compromise in terms of surgical realism, availability, didactic value, and cost/time effectiveness. We believe it is a powerful and effective tool with high potential for improving surgical education and training.

## Backgrounds

The introduction of microvascular free flaps into plastic and reconstructive surgery has greatly expanded reconstructive options. Many different forms of microvascular flaps can be harvested to suit the surgeon’s and patient’s needs and provide different tissue qualities. The workhorse flap in the field of free flap surgery has been the radial forearm free flap (RFF) since its invention in 1978 and first publication in 1981 [1]. Since its initial description, it has been widely used by the global surgical community to address a variety of reconstructive challenges. In head and neck reconstructive surgery, the popularity of fasciocutaneous flaps are largely due to their advantages, such as consistent anatomy combined with a long and high-caliber vascular pedicle. Especially for intraoral reconstruction, the excellent pliability and thinness of the flap allow sufficient reconstruction of the various tissues of the oral cavity [2]. However, one of the main advantages is the relative ease of flap elevation compared to that of other microvascular flaps, which is why it is often considered suitable for beginners in free flap surgery. Despite being considered a novice flap, there are few teaching opportunities for surgeons during the learning process. The concise teaching of free flap surgery techniques to inexperienced learners is a challenging task, as complex anatomical structures and their three-dimensional relationships need to be illustrated in combination with the necessary operative steps necessary to dissect and successfully harvest the flap. The workgroup previously focused on improving this task by developing a realistic anatomical model that can simulate the RFF flap raising process [3]. This model provides an easily accessible and cost-effective way to illustrate the relevant process and anatomy, but it cannot address the issues of flap design and tissue dissection, nor can it provide realistic microvascular training. In the published literature, many efforts have been made by the global surgical community to improve the teaching of free flap surgery.

Various live animal models have been described for the surgical process of flap harvesting. In vivo porcine models have been utilized to simulate a life-like flap dissection process, including the deep inferior epigastric perforator (DIEP) flap or the transverse musculocutaneous gracilis (TMG) flap, the superior gluteal artery perforator (SGAP) flap or other forms of perforator flaps [4–6]. These models can provide realistic experience but are not easily accessible for routine general training due to the use of large live animal models and their associated drawbacks of high cost, limited availability, and ethical concerns. The other main form of free flap training is usually performed on human cadaveric specimens. This structured training can also provide a very effective form of training [7, 8] but also has many of the disadvantages mentioned above. In general, the training of microvascular free flap surgery on living porcine and human cadaver models is usually costly, time-consuming, highly organized, and therefore ineffective, especially in early learning stages.

To improve the currently available methods for teaching microvascular free flap surgery, our workgroup attempted to develop a cost-effective and widely available training model with a sufficient level of realism for the early learning curve of prospective reconstructive surgeons.

## Materials and methods

### Development of a microvascular fasciocutaneous free flap model

**Fig. 1:**
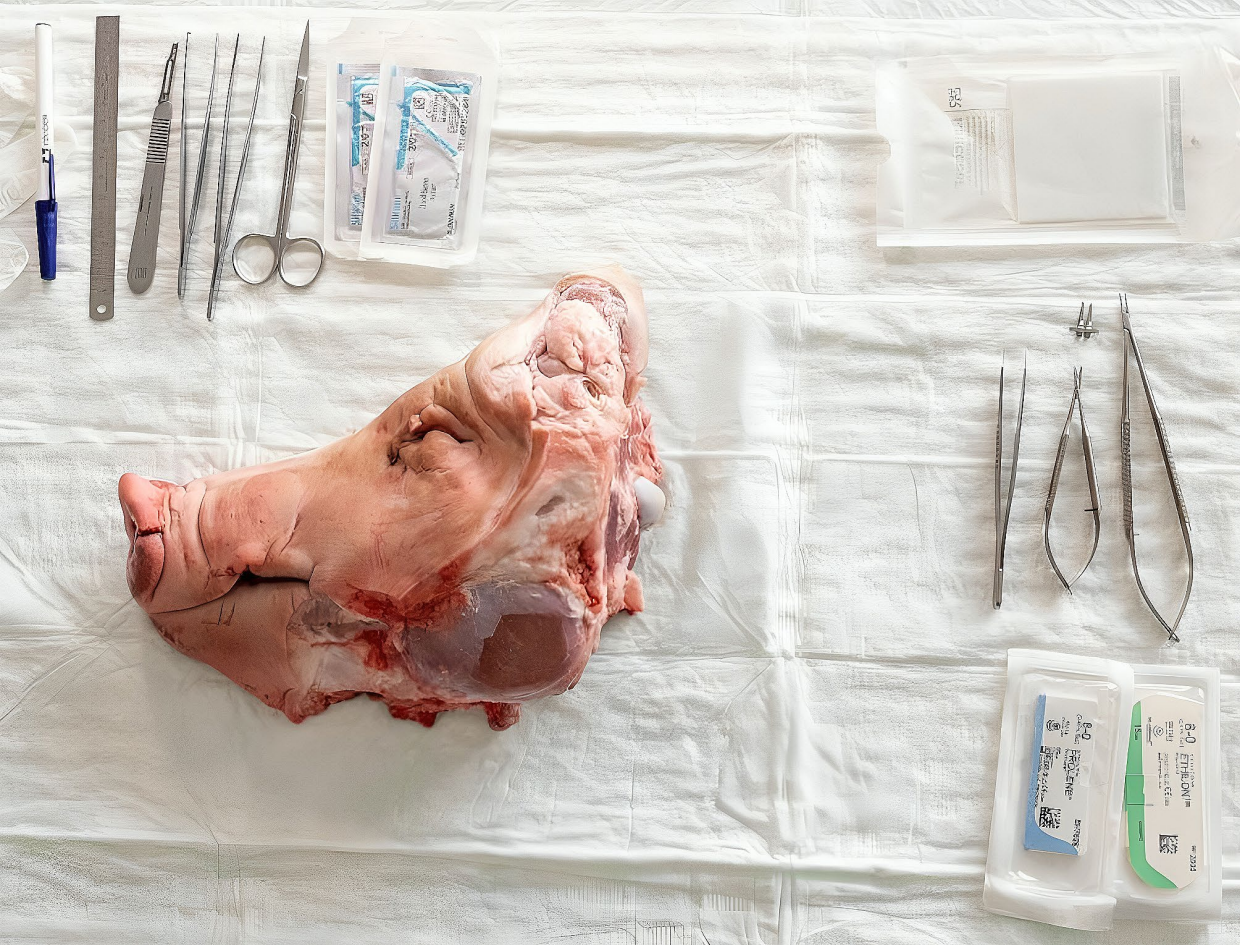
The porcine head halves. The porcine head halves with standard surgical instruments and sutures

In medical education, particularly in the surgical training of dental students, porcine head halves are commonly used for training, e.g., mucoperiostal flap preparations and tooth extractions. These animal parts accrue in large quantities by the food industry for daily meat production. The used porcine head halves are stripped of parts relevant to food production, but most of the anatomical structures necessary for medical training are still present [Fig. 1]. We attempted to assess the possibility of obtaining a fasciocutaneous microvascular free flap with these residual materials. In this region, we searched for vessels of sufficient caliber and a suitable perforator skin island for flap design. The explorations performed showed a continuous presence of vessels, corresponding to the human facial vessels in the porcine offal (facial vein). We therefore undertook an anatomical study to evaluate the vascular course of porcine facial vessels in relation to existing facial landmarks with regard to microvascular free flap harvesting.

### Study design and data acquisition

Porcine material was obtained from a local meat processing facility. The facility confirmed in writing beforehand that no animals had to be sacrificed specifically for the study and that the pig heads were a product that is routinely generated in large quantities during daily meat production. Pig heads are produced in quantities of several thousand pieces per day, as pork is considered a staple food in Germany. One half of a porcine head was required for each flap exercise. The porcine material was obtained freshly from a local meat processing facility (Contifleisch GmbH, Erlangen, Germany). The head halves could be stored for up to three days under standard conditions in a regular refrigerator without experiencing any significant loss in tissue quality, and no additional preparations were necessary.

### Study sample

The study involved 51 porcine head halves that were acquired after suturing training courses for medical and dental students [Fig. 1]. The collection was carried out from 01.04.2022 to 01.02.2023, and the anatomical study took place at the Department of Oral and Cranio-Maxillofacial Surgery of the Erlangen University Hospital of the Friedrich-Alexander-Universität Erlangen-Nürnberg. The study was conducted in accordance with the Declaration of Helsinki.

### Materials used

The study was conducted using porcine head halves [see Fig. 1] that were already used in medical training courses. Surgical markers were used for flap design and to indicate anatomical landmarks and measurement points. A standard surgical instrument kit was used for tissue dissection. Standard operating microscopes (Zeiss OPMI pico System with table mount by Carl Zeiss AG, Oberkochen, Germany) were utilized for microvascular anastomosis in our in-house educational skills lab (“MKG Skills Lab”, Department of Oral and Cranio-Maxillofacial Surgery of the Erlangen University Hospital, Erlangen, Germany). Standard microsurgical instruments and vessel approximators were used in conjunction with nonresorbable 8 − 0 sutures (Ethilon 8 − 0, Ethicon, Johnson & Johnson, New Brunswick, New Jersey, USA).

### Data collection methods

The relevant anatomic landmarks were indicated on the porcine head halves. The line connecting the medial corner of the eye to the lateral corner of the mouth was then marked and used as the main orientation for measurements. After the connecting line was marked, a sharp incision was made alongside it, and the vascular pedicle (the porcine facial vein) was prepared by surgical tissue dissection. The location of the vascular pedicle was photographed, and the distance along the connecting line was measured in relation to the medial angle of the eye. The entire course of the vascular pedicle was then traced in a proximal direction to the end of the porcine model in the cervical region.

### Variables

The distance of the vascular pedicle from the medial corner of the eye on the connection line to the lateral corner of the mouth was measured. After dissection of the vascular pedicle, the angle to the connecting line was observed.

### Data analysis and statistical calculations

Statistical analysis was performed on the anatomical variations in the position of the vascular pedicle. Particular attention was given to the reliability of accurate localization of the underlying vascular pedicle using only external skin measurements. Statistical analysis of the baseline data was performed using SPSS 24.0.0.2 software (IBM SPSS Statistics, IBM Corporation, Chicago, IL, 1989, 2016). A p-value < 0.05 was considered to indicate statistical significance.

## Results

### The final microvascular free flap model

**Fig. 2:**
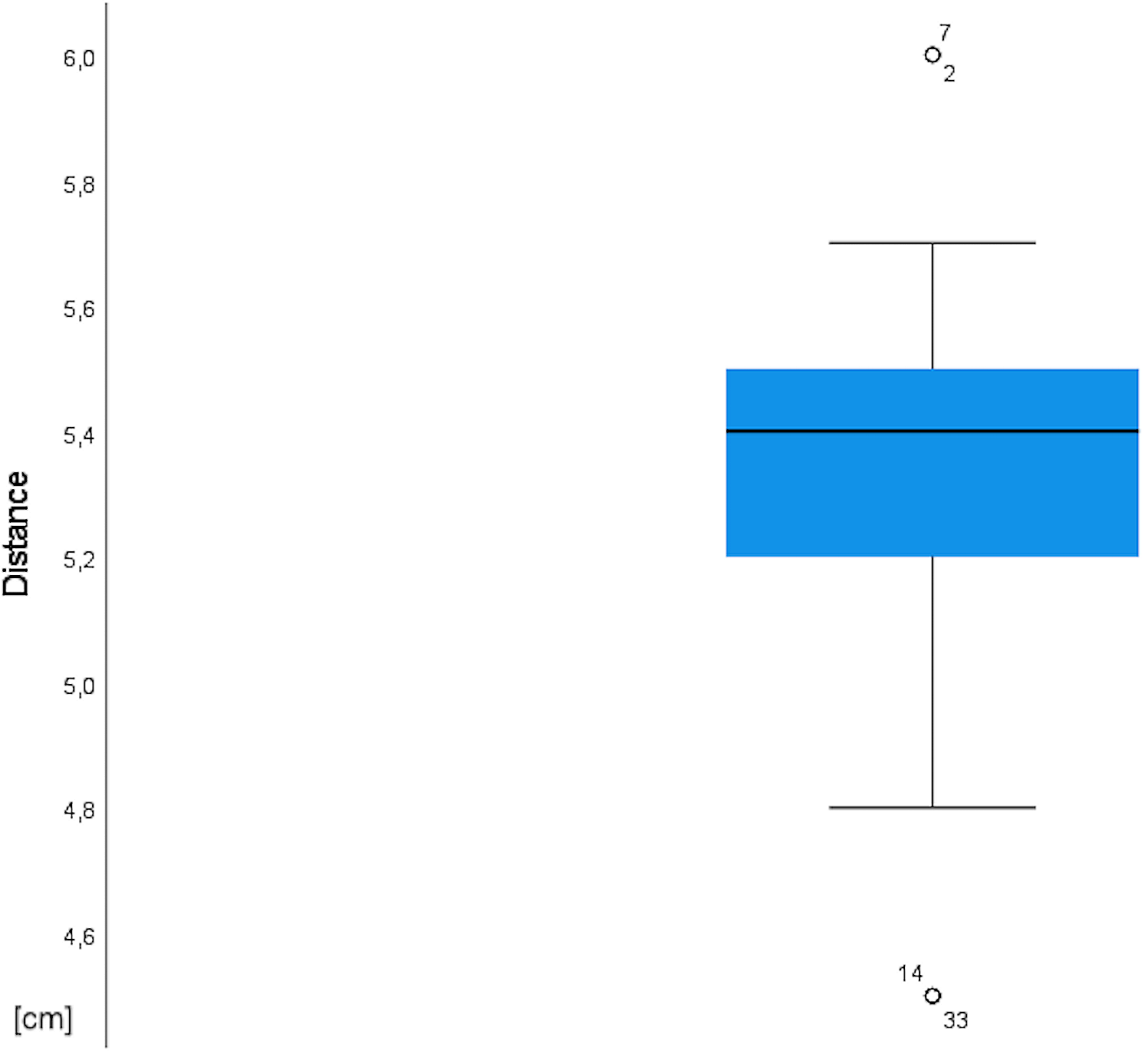
The localization of the vascular pedicle. Results of the distance of the vascular pedicle from the medial corner of the eye on the connecting line to the lateral corner of the mouth displayed as a box plot

**Fig. 3:**
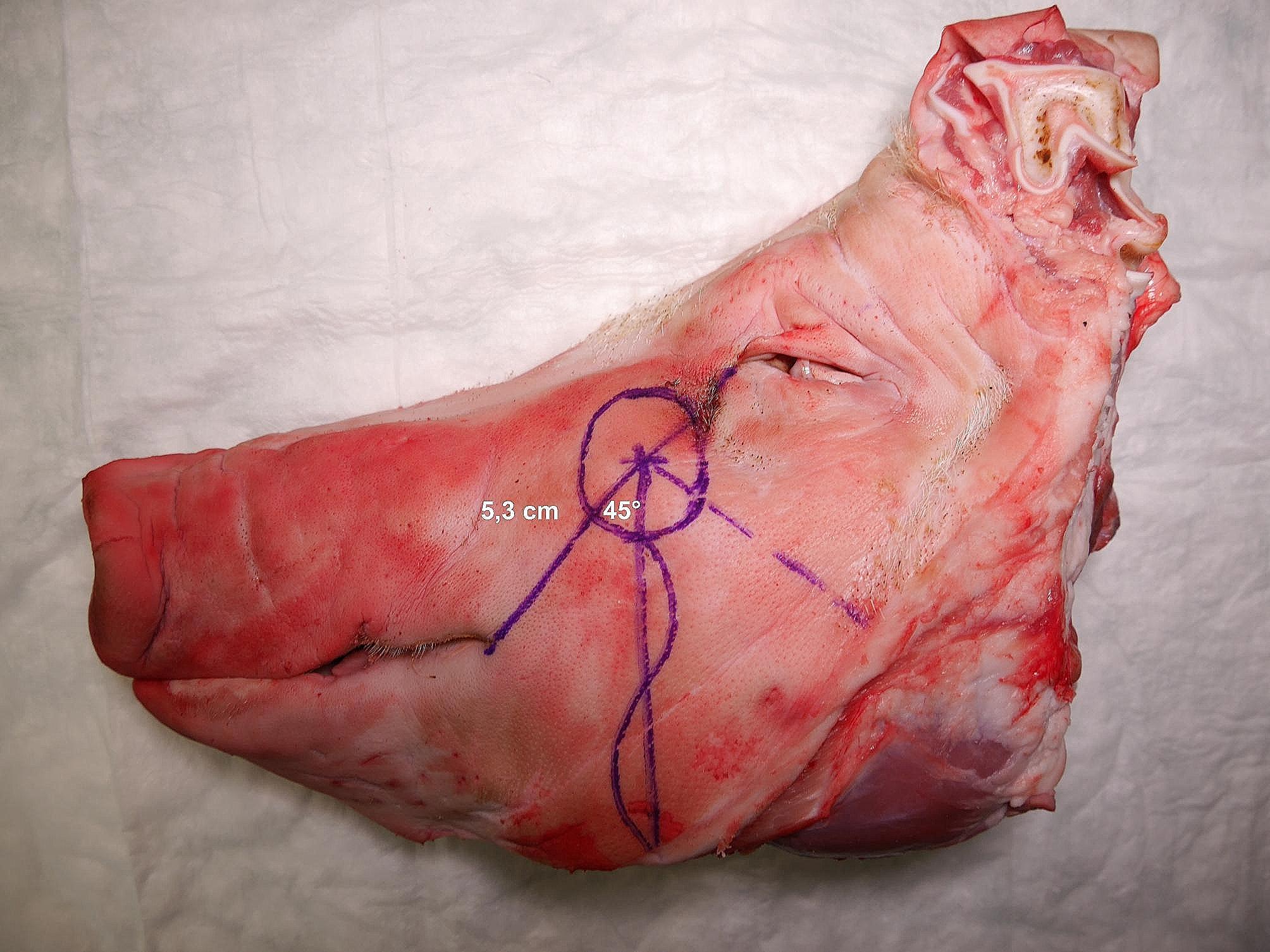
Anatomic landmarks for free flap harvesting. The indicated anatomical landmarks and vessel course prior to dissection. The presumed course of the vascular pedicle is marked in red

**Fig. 4:**
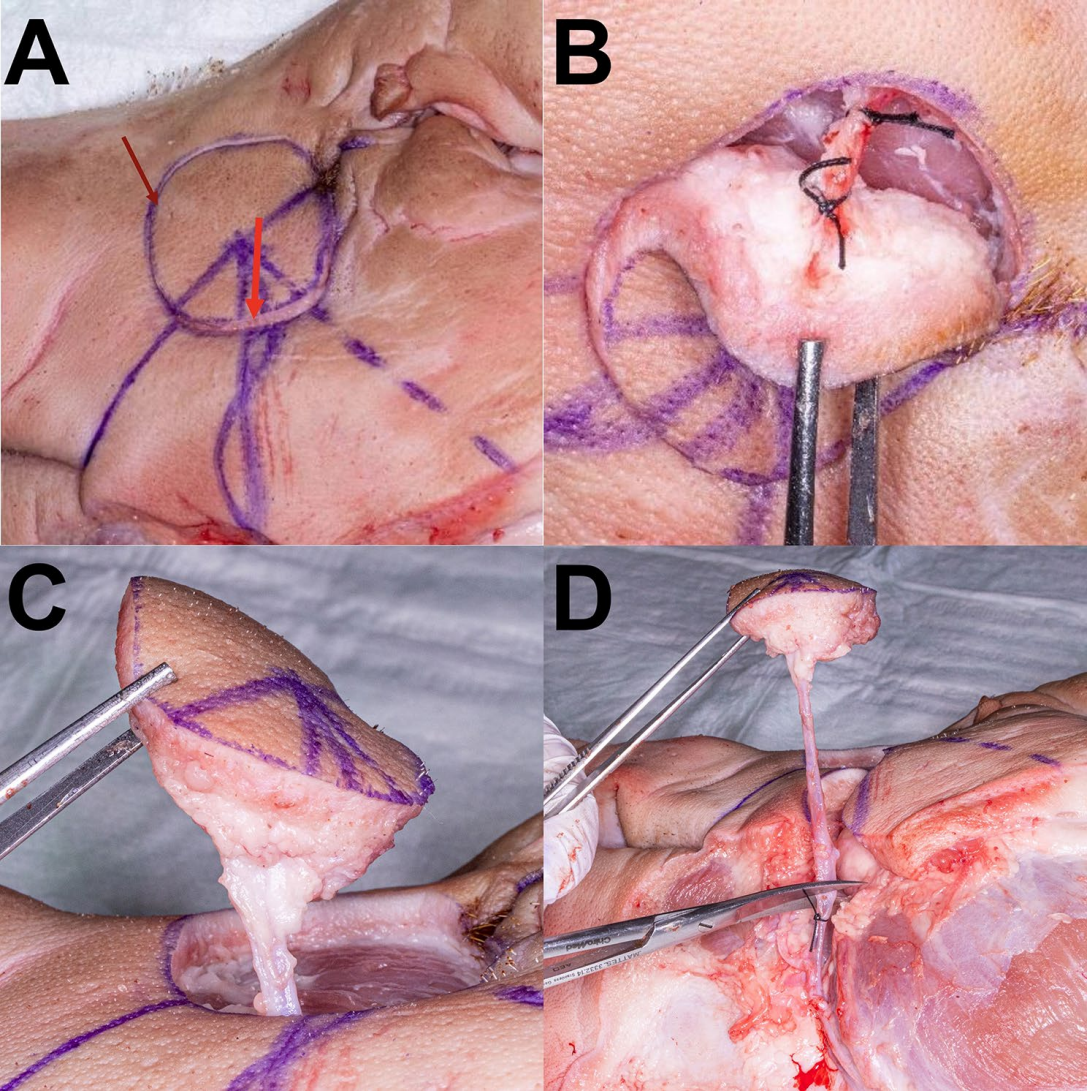
The surgical flap raising procedure. The detailed process of the free flap harvesting. **A**: The finished markings with the desired extension of skin island (steps I-III). The circular incision is made around the skin island is performed (dark red arrow) and the proximal part of the vascular pedicle is located via surgical dissection (light red arrow) **B**: The ligation of the distal part of the vascular pedicle (step IV) **C**: Further dissection of the flap from the surrounding tissue and elevation from the underlying muscles (step V) **D**: The vascular pedicle is fully exposed, the proximal part of the pedicle is ligated and the free flap is fully elevated

**Fig. 5:**
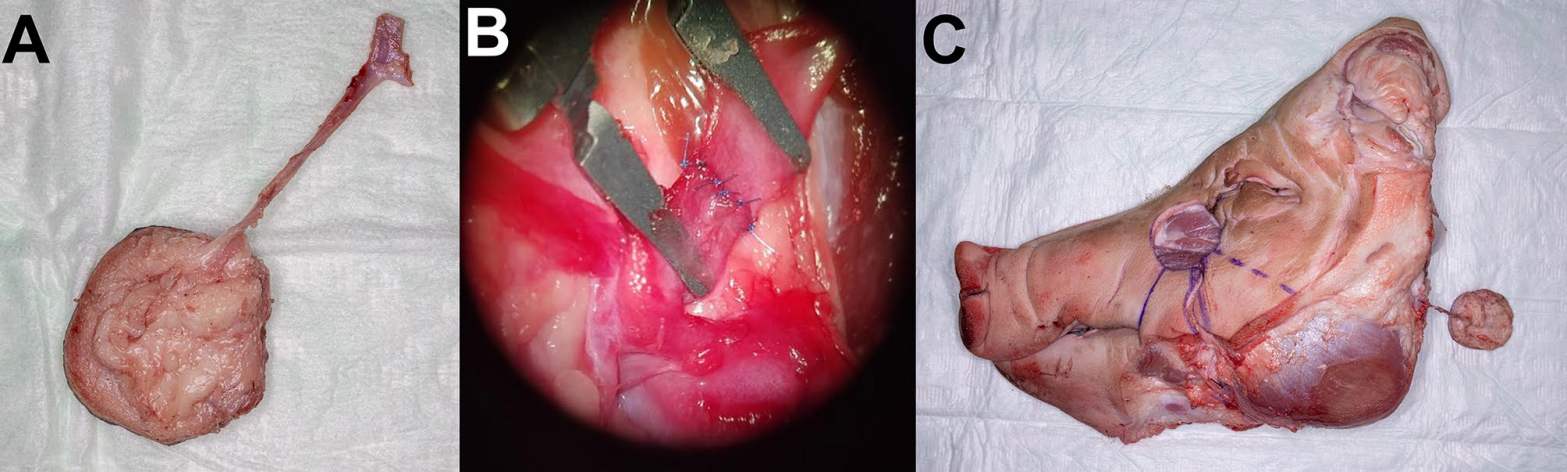
Microvascular anastomosis of the free flap. Microvascular anastomosis is performed after competition of the free flap harvest **A**: The free flap is transferred to the desired location and the vascular pedicle is placed on the recipient vessels on the neck **B**: Microvascular anastomosis in the vessel approximator under the surgical microscope **C**: Completed microvascular anastomosis to the local neck vessels

In total, 51 porcine head halves were examined in the anatomical study [Fig. 1]. The presence of an anatomically very consistent vessel, suitable for mimicking a vascular pedicle was observed in all specimens by surgical dissection. This single vessel was identified as the porcine facial vein, as the facial artery’s course was too deep, partly within the bone of the skull, preventing its inclusion in the pedicle. The vascular pedicle of the model could be harvested with a length ranging from 10 to 15 cm and featured a vessel diameter between 2 and 3 mm. These values are comparable to those of human fasciocutaneous free flaps [2]. The course of the vessel was consistently observed at a mean distance of 5.3 cm from the lateral corner of the mouth on the connecting line to the medial corner of the eye [Table 1]. The vascular pedicle was accurately identified at the specified distance, facilitated by the low standard deviation of 0.3268 cm across all porcine head halves [Fig. 2]. Its termination was discernible at the lower border of the porcine models within the neck region. The observation revealed that the pedicle consistently followed a downward angle of 45° in a clockwise direction from the mouth-eye connecting line [Fig. 3]. These consistent results provide a reliable basis for planning microvascular free flap procedures, where a stable vascular anatomy is imperative for success. To enhance comprehension, the process of free flap harvesting in the model has been methodically outlined into six key steps, akin to the haptic anatomical model for radial forearm free flap harvesting previously developed by the workgroup [3] [Table 2; Fig. 4]. First, the connecting line between the medial corner of the eye and the lateral corner of the mouth is marked on the porcine head halves (Step I). The location of the vascular pedicle was then marked on the connecting line 5.3 cm away from the lateral corner of the mouth (Step II). The vascular pedicle is located directly below this marked position. To design the skin island of the fasciocutaneous flap, it is necessary to further indicate the course of the pedicle. To do this, a perpendicular must be drawn posterior to the vessel location mark, and the anterior angle bisector must be constructed. This anterior angle bisector, which forms the connecting line at an angle of 45° downward in the clockwise direction from the interconnecting line, indicates the course of the vascular pedicle (Step III). With the course of the vascular pedicle now visualized on the outer skin, the desired extension of the flap island can be designed. After marking the extension, a sharp circular incision was made around the skin island. Surgical dissection is then performed to locate the vascular pedicle at the proximal and distal flap margins. The distal part of the vascular pedicle was cut and ligated (Step IV). Tissue dissection is performed along the borders of the skin island until the underlying muscle plane is reached. The flap is then dissected further until it is completely elevated from the muscular plane, and the overlying fascia is integrated into the flap (step V). Once the skin island has been surgically elevated, a wave-shaped incision is made at the inferior border of the skin island, following the previously marked vessel course. The vascular pedicle is traced and exposed along its proximal course to create a sufficient pedicle length (Step 6). The flap is now completely harvested and can be transferred for microvascular anastomosis [Fig. 4]. The fasciocutaneous free flap can now be freely placed in any part of the porcine head halves, e.g., in a second created defect. The inferior border of the head halves usually offers many local recipient vessels of varying degrees of difficulty for further microvascular anastomosis. We recommend continuing dissection of the external carotid artery at the dorsal border of the flap and performing microvascular anastomosis at this point. A long pedicle length provides many options for later placement of the skin island. Microvascular anastomosis can be performed as desired; in our cases, after vessel preparation, anastomosis was performed using the end-to-end technique with 8 − 0 nonresorbable suture material in the vessel approximator between the facial vein and the external carotid artery or its side branches, e.g., the lingual artery [Fig. 5].

**Table 1:** Localization of the vascular pedicle

		Overall (*n* = 51)
Distance from the lateral corner of the mouth on the connecting line to the medial corner of the eye	mean (median)	5.32 (5,4)
	range (min-max)	1.5 (4.5-6.0)
	SD	0.3268

Results of the anatomical study of the localization of the vascular pedicle

**Table 2:** Surgical steps for porcine facial free flap harvesting

I.	Indicate the connecting line between the medial corner of the eye and the lateral corner of the mouth.
II.	Mark the location of the vascular pedicle at 5.3 cm away from the lateral corner of the mouth on the connecting line to the medial corner of the eye
III.	Raise a perpendicular on the above mark in posterior direction and construct the anterior angle bisector to indicate the course of the vascular pedicle. Mark the desired extension of the skin island on the course of the vascular pedicle.
IV.	Perform the circular incision around the skin island and continue with tissue dissection to locate the vascular pedicle on the proximal and distal flap border. The distal end of the vascular pedicle is cut and ligated.
V.	The flap is further dissected from the surrounding tissue and is elevated from the underlying muscles as basal border.
VI.	A wave-shaped incision is performed, and the vascular pedicle is exposed and the free flap is fully elevated.

## Discussion

The clear and effective teaching of surgical procedures remains a challenging task for the medical community. Complex surgical procedures are difficult to teach solely in the operating theatre, and a great deal of preparatory work is needed to enable the student or resident to follow and understand the complex procedures effectively. In addition, technical skills are needed to perform the required surgical task. Even today, surgical procedures are often learned through theoretical preparation by reading relevant publications or research articles, followed by step-by-step practical training in the operating theatre [9]. Unfortunately, training in the operating theatre is time consuming [10] and even more so without prior preparation of the trainee. In recent years, numerous factors have significantly influenced surgical teaching and training. These include reduced working hours, economic pressures, and the imperative to optimize the use of operating theaters while ensuring patient safety. However, the most notable impact has been the recent COVID-19 pandemic. The disruptions it has caused have been extensive, affecting didactic sessions, research activities, and surgical training programs, thereby presenting additional challenges [11]. The pandemic’s contact restrictions prompted not only changes in medical teaching methodologies but also the recognition of the various advantages offered by simulation training. Consequently, there is a growing advocacy for a shift in learning and teaching approaches with an increased integration of simulation. Simulation facilitates the training of technical, cognitive, and behavioral skills in an immersive and realistic manner, providing a patient-safe environment for acquiring a wide range of proficiencies [12]. The increasing integration of simulation models has underscored the importance of utilizing diverse tools for assessing relevant skill proficiencies [13]. Over the years, the medical community has attempted to improve the teaching and learning of surgical procedures using various forms of simulation. A study by *Villanueva et al.*. used a combination of simulators such as full manikins, part-task trainers or tabletop models and virtual reality systems for cardiothoracic surgical education and training. Various procedures could be effectively simulated using the above simulators, and simulation was shown to improve learning and trainee performance [14]. Another form of simulation in surgical education, especially in more specialized training for residents, is the use of animal models. These can be only parts of the deceased animal, as shown for cricothyrotomy trainers [15] with porcine trachea, or the popular use of fresh chicken leg for microvascular anastomosis training [16–19]. Another option is the use of whole euthanized animals, such as for aortic anastomosis training [20], or live animals, as is often the case with rats, which are commonly used for various microvascular training and flap harvesting techniques [21–23]. Furthermore, the use of human cadavers for training is often advocated, as this is the only way to work on regular human anatomy and tissues apart from living patients [24]. All of these models can effectively mimic real-life situations in different ways, in a safe environment without the risk of harm to the patient and without time pressure, where mistakes can be made without the consequences of the clinical situation. In general, animal models can provide a good solution in terms of tissue realism, cost-effectiveness, and availability, but a drawback of models based on live animals is the sacrifice of live animals solely for training purposes, which conflicts with animal welfare ethics. In the field of microvascular free flap surgery, many of the above models exist to illustrate the operative process, but there is still a need for a model that allows training of the entire flap harvesting and microvascular transfer process while being cost-effective, easily accessible, and free of ethical concerns. As no such sufficiently realistic model has been previously described, we developed a porcine free flap surgery training model. We identified porcine head halves as the most suitable in terms of availability, cost, and degree of realism. The model relies solely on meat production waste that would otherwise be discarded as an offal. This makes the model ethically acceptable from an animal welfare perspective, as no additional animals need to be harmed for training and education purposes. The use of split porcine head halves is not new to surgical education. For example, *Kersey et al.* used porcine head halves to train oculoplastic procedures [25], and *Kuwahara et al.* used this model to practice cutaneous surgery techniques [26]. We attempted to adapt this well-established surgical model to the field of free flap teaching, but for this purpose, the validation of a suitable vascular anatomy had to be evaluated. The present study, which included the dissection of 51 porcine head halves, revealed a reliable vascular anatomy with the possibility of sufficiently realistic fasciocutaneous flap harvesting [Table 1]. After evaluating the sufficient anatomical requirements, we successfully designed and implemented a surgical protocol for fasciocutaneous free flap training and microvascular anastomosis [Table 2]. The flap harvesting process is divided into 6 key steps that are easy to understand and follow. The surgical procedure is very similar to real surgery, such as radial forearm free flap harvesting. The flap can then be freely transferred to the desired defect site, and microvascular anastomosis can be trained on a variety of local recipient vessels in the cervical region. The quality of the microvascular anastomosis performed during exercise can be assessed through direct surgical incision of the vessel, followed by clinical inspection of the sutures. This inspection should focus on evaluating the depth and even distribution of the sutures, as well as identifying any potential through-stitches. Additionally, vessel patency can be evaluated by perfusing the vessels using various devices, if desired [27, 28]. All the highlighted surgical techniques are feasible through a single-surgeon approach. However, based on our experience, adopting an operator-assistant approach further amplifies the didactic benefits, particularly during microvascular anastomosis training. The model presented could serve as an initial practical step in the process of mastering radial forearm free flaps. The educational process can begin with the conventional study of relevant anatomy from textbooks, augmented by the 3D anatomy provided by the haptic radial forearm flap model. Subsequently, the essential surgical steps of flap harvesting can be systematically reviewed on the presented porcine model, coupled with microvascular training. By mastering these fundamental technical aspects, training can progress to advanced stages, involving practice on live animal models or gradually performing more extensive portions of the operation on actual patients under the close supervision of an experienced surgeon. A major advantage of the model is that it is readily available in large quantities at a low cost, making it suitable for large group hands-on workshops and providing realistic access to free flap techniques for a wider audience. No preparation is needed for the porcine head halves, which can be stored under regular refrigerator conditions. In addition, the only necessary items are standard surgical and microsurgical instruments, suture materials, and a standard microscope or magnifying glasses. The illustrated model can be integrated into the training of residents or advanced medical students and can be combined with other surgical simulators, such as anatomical models or 3D simulations, to further enhance the didactic value. It provides an effective and valuable method of initial preparation at an individual speed and intensity before taking the first steps in the operating theatre, where time for surgical teaching is often limited. The process of flap raising and of the anatomical structures involved can be experienced first, and surgical techniques such as suturing, tissue dissection and microvascular surgery can be trained on real tissue. One limitation of the model is its restriction to harvesting a single free flap per porcine specimen. However, the surplus animal material can be effectively utilized for supplementary suture training or for practicing local flap raising and defect covering exercises. Another limitation of the model lies in its mimicking of a vascular pedicle, which features only one venous vessel. Although comparable in terms of diameter and pedicle length to those of a radial forearm free flap, the typical configuration of an artery with two accompanying veins is not anatomically present in the porcine model. The realism of the presented model is inherently constrained by its status as a nonliving animal model, devoid of functioning vessels with consistent blood flow. Consequently, surgical techniques such as perforator identification, subsequent preservation, and surgical hemostasis cannot be accurately simulated. In addition to microvascular anastomosis, the future integration of diverse perfusion methods could significantly enhance the learning experience and augment the didactic benefits [27, 28]. In our department, the model is nevertheless being incorporated into the routine training of residents in reconstructive surgery. A clear drawback of the present research is the lack of verification of the proposed effectiveness, which will be investigated by further evaluations in the future. In addition, further research is certainly needed into how training with these specific and other analogous models translates into improved performance in the operating theatre.

## Conclusions

Currently, surgical education is rapidly evolving, facing new challenges and benefiting from various developments. Especially in the field of microvascular free flap surgery, improvements are needed to effectively teach the relevant surgical procedures. Therefore, we developed and implemented a training model based on porcine head halves, which can effectively illustrate the entire surgical process of realistic microvascular free flap surgery. The developed fasciocutaneous free flap training model represents a reasonable compromise in terms of surgical realism, availability, didactic value and cost/time effectiveness. We believe it is a powerful and effective tool with high potential for improving surgical education and training.

## Data Availability

Most of the data generated or analyzed are included in the article. The remaining datasets used and/or analysed during the current study are available from the corresponding author upon request.
